# Selection for evasive mimicry imposed by an arthropod predator

**DOI:** 10.1098/rsbl.2023.0461

**Published:** 2024-01-03

**Authors:** Karl Loeffler-Henry, Thomas N. Sherratt

**Affiliations:** Department of Biology, Carleton University, Ottawa, Ontario, Canada K1S 5B6

**Keywords:** evasive mimicry, Chinese mantid, calyptrate fly, anti-predator defence, Batesian mimicry, insect learning

## Abstract

It has long been hypothesized that a species that is relatively easy to catch by predators may face selection to resemble a species that is harder to catch. Several experiments using avian predators have since supported this ‘evasive mimicry’ hypothesis. However, the sudden movement of artificial evasive prey in each of the above experiments may have startled the predators, generating an avoidance response unrelated to difficulty of capture. Additionally in the above experiments the catchability of prey was all or nothing, while in nature predators may occasionally catch evasive prey or fail to catch slower species, which might inhibit learning. Here, using mantids as predators, we conducted an experimental test of the evasive mimicry hypothesis that circumvents these limitations, using live painted calyptrate flies with modified evasive capabilities as prey. We found that mantids readily learned to avoid pursuing the more evasive prey types. Warning signals based on evasiveness and their associated mimicry may be widespread phenomena in nature. These findings not only further support its plausibility but demonstrate that even arthropod predators can select for it.

## 1. Introduction

Classical Batesian mimicry arises when a palatable species (the mimic) evolves to resemble an unpalatable species (the model). This resemblance is selected for because mimics, especially when rare, will tend to gain protection from predators [1,2]. However, predators may also learn to avoid pursuing species that are sufficiently difficult to catch that they are not profitable to chase. If this occurs, then one would expect less evasive species may evolve to resemble a more evasive model, resulting in selection for Batesian evasive mimicry [3,4].

Over the years, several ‘proof of concept’ experiments have been performed to test whether avian predators could impose selection for Batesian evasive mimicry. Gibson [5,6] presented caged passerines with edible targets on platforms that could be tilted when approached, denying access to the targets. Initially, targets of some colours were rendered unobtainable, while the birds were allowed access to others. After several days training, birds were once again presented with the targets, but all platforms were left stationary. At this point, the birds demonstrated significant reluctance to pursue target colours that had previously been evasive. Hancox & Allen [7] performed a related experiment this time using free ranging birds and two different coloured balls of dough, with one colour rapidly withdrawn to simulate an evasive model. At the start and end of each of the experiment ‘control’ presentations were performed in which neither bait was withdrawn. The authors found that wild birds rapidly learned to focus on the non-evasive prey and could reverse their preferences accordingly.

Most recently, Páez *et al*. [8] experimentally compared the rate at which birds learn to avoid evasive prey compared to distasteful prey. This was done by presenting caged passerines with sunflower seeds placed on printed silhouettes of butterfly wings that varied in chromatic resemblance to different species of *Adelpha* butterfly that may serve as agile models in neotropical evasive mimicry rings. Prey were rendered evasive by placing them on a track that could be rapidly withdrawn. The authors found that learned avoidance was more rapid for evasive than distasteful prey. A follow-up experiment showed how birds could use the shape of butterfly wing tails as an indicator of evasiveness [9].

While the above experiments have provided important empirical evidence of selection for evasive mimicry, they share some fundamental limitations that need to be addressed. First, in all of these experiments the evasive prey were impossible to catch while the non-evasive prey had no means to escape whatsoever. This may represent rather an extreme test of the concept, because it is likely that wild predators will sometimes successfully catch the more evasive model and/or fail to catch the slower mimic. Secondly, and more importantly, the artificial nature of the evasive prey's escape mechanisms may have confounded the findings of these experiments. This is because a sudden movement by the prey may elicit a startle response in avian predators [10–12] that could result in aversions based not on the prey type's evasiveness, but on the surprise they elicit.

Here we address the above limitations through a laboratory experiment designed to determine if variation in the escape abilities of calyptrate flies is sufficient to cause selection for mimicry in less evasive prey. Calyptrate muscoid flies (Diptera: Schizophora) appear to be likely models for Batesian evasive mimicry as they are both abundant in most terrestrial ecosystems and highly evasive [13–15]. By cutting the wings of flies with certain colour patterns and not cutting those with an alternative colour, we manipulated their evasiveness directly in a manner that did not involve the sudden withdrawal of prey and allowed evasive prey to occasionally be captured.

Our experiments were conducted using arthropod rather than avian predators, namely mantids. Arthropods, ranging from dragonflies to spiders, are widespread, diverse and voracious insectivores that have long been considered candidate agents of selection on warning signals and mimicry [1,16–20], in part because they have been shown to exhibit learned aversion [21,22].

Notably, Maldonado [23] showed that after several unsuccessful attacks towards stimuli that were impossible to catch, mantids (*Stagmatoptera biocellata*) showed a marked decrease in responsiveness. To our knowledge however, this is the first experiment to test directly whether arthropods might learn to discriminate among prey based on signals that indicate difficulty of capture.

## 2. Methods

See electronic supplementary material for details of how the mantids (*Tenodera sinensis*) and wild caught muscoid flies were acquired and prepared for each phase of the experiment.

### (a) Training phase

The training phase of our experiment involved repeatedly presenting two similar-sized flies, one with thorax painted black and one with thorax painted white, to individual mantids. We chose these shades to maximize the luminance contrast and because of the limited colour vision of mantids [24]. The presentations (seven in total) were made every second day over a period of two weeks. The two presented flies were clipped and painted as part of a 2 (black B or white W) × 2 (clipped C or not clipped N) treatment design i.e. only the white fly was clipped (BN/WC; treatment 1), only the black fly was clipped (BC/WN; treatment 2), both flies were clipped (BC/WC; treatment 3), neither fly was clipped (BN/WN; treatment 4). Each mantid only experienced a single training treatment and there were 20 mantids per treatment.

Presentations of flies to mantids took place in sequential order based on treatment, so that flies were first given to a mantid in treatment 1, then a mantid in treatment 2, then 3 and 4. After set-up, the flies and mantids were checked every 20 min over a 3 h duration. Once a mantid had caught one fly, the remaining fly was removed. If no flies were caught after 3 h both were removed. Since it takes mantids of the size (L3–L4) used in this experiment more than 20 min to consume a fly, the mantids were never able to consume both flies between consecutive checks. Note that neither failed capture attempts nor the precise timing of successful captures were recorded in the training phase, with observations limited to the fly type that was successfully caught.

### (b) Test phase

The test phase was conducted once for each mantid and involved presenting unclipped (BN/WN) yet tethered flies and observing which colour of fly, if any, was first attacked. The mantids were tested following the same sequential order of treatment as above and monitored in the same manner as in the training phase.

### (c) Statistics

All of our statistical models were fitted in R version 4.2.1. To test whether flies of a given colour were more likely to be successfully captured than the alternate colour during the course of the training phase we compared the summed frequency of successful captures of flies of the two colours of within any given treatment using a Wilcoxon signed rank (WSR) test, with pairing based on the individual mantid.

To test whether the treatment regime affected the choice of fly attacked in the test phase we first reduced the data to cases where a fly had been attacked by a mantid (72 of 80 cases). Since only one data point was observed per individual mantid we fitted a generalized linear model (glm) to this binary response (black/white fly first attacked) assuming a logit transform, with training treatment as a categorical predictor. The importance of training treatment in explaining variation in the type of fly attacked in the test was assessed by fitting an intercept only (null) model and comparing the two models using a log likelihood ratio test (LRT). Based on a significant effect of treatment, differences in the log odds of attacking black flies among the possible training treatment combinations were assessed using the glht function of the multcomp package. To test whether white flies are attacked significantly more or less than black flies in the test phase, we performed a binomial sign test for each treatment.

## 3. Results

**Figure 1.**
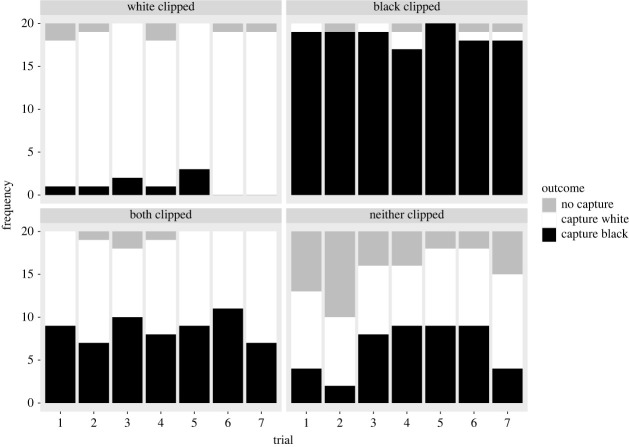
The frequency of outcomes (successfully capture white flies, successfully capture black flies or do not capture any fly) observed when mantids were presented with a choice of flies over seven consecutive trials in the training phase (20 mantids per treatment). Note that when neither fly had its wings clipped, there was a higher incidence of no fly being caught at the end of the observation period (3 h).

Over the course of training in the BN/WC treatment, white flies (which had been clipped) were successfully caught more frequently than black flies (which had not been clipped) (WSR *V* = 210, *p* < 0.0001). Likewise, over the course of training in the BC/WN treatment, black flies (which had been clipped) were successfully caught more frequently than white flies (which had not been clipped) (WSR, *V* = 0, *p* < 0.0001). By contrast, there was no significant difference in the successful capture distributions on flies of the two colours in the BC/WC treatment when both colours of fly had been clipped (WSR *V* = 127.5, *p* = 0.1925) or in the BN/WN treatment when neither colour of fly had been clipped (WSR *V* = 104, *p* = 0.0628; figure 1).

**Figure 2.**
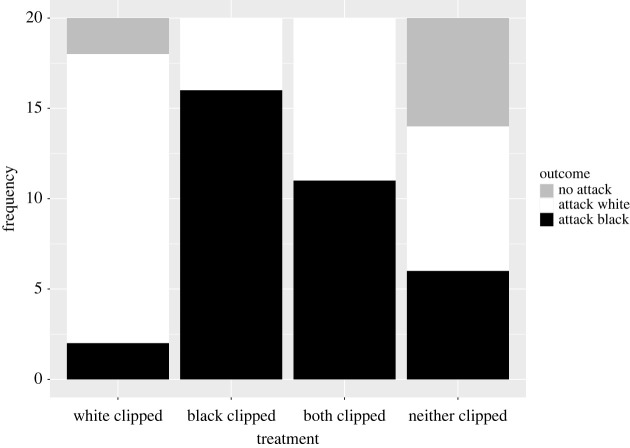
The frequency of outcomes (attack white flies, attack black flies or do not attack any fly) observed in the test phase (20 mantids per treatment).

In the test phase, involving a presentation of a single black and single white fly that had not been clipped, the log odds of attacking black versus white flies differed significantly among treatments (LRT $\chi_{3}^{2}=20.537$, *p* = 0.0001). Mantids that had experienced training treatment 2 (BC/WN) had a significantly higher odds ratio of attacking a black fly in the test phase than mantids in the BN/WC treatment, where white flies had been clipped (z = 3.705, *p* = 0.0011). Likewise, mantids trained in the BC/WC treatment (both types of fly clipped) had a marginally significant higher likelihood of attacking black flies in the test phase than mantids trained in treatment 1 (BN/WC) (z = 2.608, *p* = 0.0443; figure 2).

In the test phase, significantly more white flies were caught (16) than black flies (2) by mantids trained in the BN/WC treatment (sign test, *p* = 0.0013). Significantly fewer white flies were caught (4) than black flies (16) by mantids trained in the BC/WN treatment (sign test, *p* = 0.0118). There was no evidence that the attack rates on white and black flies were different for mantids trained in the BC/WC (sign test, *p* = 0.8238) and BN/WN (sign test, *p* = 0.7905) treatments.

## 4. Discussion

The findings from the test phase of our experiment provide clear evidence that mantids can learn to attack prey with signals that they had previously found easier to catch. This suggests that, rather than attempting to pursue prey to directly test their escape abilities, the mantids can learn to use colour as a signal of escape ability. As such, any slow-moving mimic that looked like an evasive model may gain a selective advantage over non-mimics (Batesian evasive mimicry).

To our knowledge, our experiment is the first to test the evasive mimicry hypothesis using live prey specifically modified to have different evasive capabilities, rather than using some form of apparatus to simulate evasiveness. Moreover, since our evasive models can occasionally be caught, our experiments are not only more natural, but also provide a stronger test of the concept, since capturing evasive prey is likely to inhibit learning. Our experimental prey were calyptrate flies. These flies are generally regarded as hard to catch [13] and may well be models to evasive mimics [15,25]. Indeed, many arthropods appear to have evolved a resemblance of calyptrates, including 70 species of beetles, the jumping spider *Scoturius dipterioides*, and moths from the *Macrocilix* genus, which possess markings on each forewing that resemble a calyptrate fly [26–29].

Our experiment asked whether arthropod predators can in principle select for evasive mimicry. Arthropods have been somewhat neglected as a selective force in the evolution of warning signals and mimicry [17,19]. In his classic work, Bates [1] reported that heliconid butterflies were not attacked by invertebrate predators including dragonflies and robberflies, despite the fact that these invertebrates were often seen attacking butterflies from other families and used these observations to infer their unpalatability. Similarly, Shelly & Pearson [30] noted that robber flies attacked chemically defended tiger beetles with orange abdomens less frequently than tiger beetles with dark abdomens, proposing that they may be partially responsible for the evolution of warning signals in this species.

Although using live prey to simulate evasive mimics and models avoids many of the potential limitations of using artificial prey, it may introduce some other confounders. First, rendering some flies non-evasive by clipping one wing may affect aspects of their behaviour beyond evasiveness. Ideally, prior to being presented to the predator, the evasive and non-evasive prey would undergo identical handling and modification. Naturally, this is not possible. However, this discrepancy in the training phase is largely addressed in the test phase, when the mantids were presented with tethered flies that had been subject to identical handling. Additionally, taxonomic heterogeneity in the wild-caught flies used in the experiment (see electronic supplementary material, methods) may have introduced biases. Although the intact-winged flies always appeared more evasive than those with clipped wings, different fly taxa may exhibit differences in caloric profitability, gustative quality and behaviour that could influence mantids' proclivity to attack them. Randomly allocating flies to treatments, along with the experiment's relatively large sample size, likely mitigated the probability of cross-treatment heterogeneity in fly taxa. Additionally, the lack of a significant difference in capture success between the different-coloured flies when they were modified in the same way (BC/WC and BN/WN treatments) suggests that any biases introduced by taxonomic heterogeneity in flies were minor.

While signals of chemical defence have been the subject of considerable research interest, signals of other defences have received comparatively little attention [2]. There appears to be a growing awareness however that prey can be unprofitable to pursue not only because they are distasteful but also because they are hard to catch [8,25]. For these reasons, highly evasive species may evolve warning signals in the mutual interests of the prey and any optimally foraging predators [4]. However, signals of evasiveness can also be mimicked by less evasive species. Batesian evasive mimicry was originally proposed as an explanation for the resemblances of several conspicuously coloured, yet edible species of African butterfly [3]. Lindroth [31] subsequently suggested that jumping flea beetles (Alticinae, Chrysomelidae) are mimicked by less evasive ground beetles (Lebia, Carabidae). Likewise, Balgooyen [32] suggested that the speckled rangeland grasshopper (*Arphia conspersa*) is an evasive mimic of the alfalfa butterfly (*Colias eurytheme*) while in flight. More recently, Pinheiro and colleagues [33,34] have drawn attention to several neotropical butterfly species that may be members of rings involving a combination of Batesian mimics (easy to catch) and Müllerian (hard to catch) models. So, examples of species copying the signals of co-occurring evasive species are widespread in nature, and our findings suggest that it is not only bird predators that can select for it.

## Data Availability

The data and code used in this study are available from the Dryad Digital Repository: https://doi.org/10.5061/dryad.s1rn8pkff [35]. Supplementary material is available online [36].
